# Distinct Clinical Phenotypes of Severe Pediatric Influenza in the Post-COVID-19 Era: Insights from a Multicenter PICU Study in Türkiye

**DOI:** 10.3390/children13010014

**Published:** 2025-12-20

**Authors:** Güntülü Şık, Servet Yüce, Tuğba Kanar, Nihal Akçay, Demet Tosun, Özge Umur, Muhterem Duyu, Ayşe Aşık, Abdulrahman Özel, Agop Çıtak

**Affiliations:** 1 Department of Pediatric Intensive Care, School of Medicine, Istanbul University-Cerrahpaşa, 34320 Istanbul, Türkiye; sare.guntulu@iuc.edu.tr (G.Ş.); tugba.kanar@iuc.edu.tr (T.K.); 2 Department of Public Health, Istanbul Provincial Health Directorate, 34122 Istanbul, Türkiye; 3 Department of Pediatric Intensive Care, Kanuni Sultan Süleyman Training and Research Hospital, University of Health Sciences, 34303 Istanbul, Türkiye; nihal.akcay@sbu.edu.tr (N.A.); demet.tosun@sbu.edu.tr (D.T.); 4 Department of Pediatric Intensive Care, Acıbadem Mehmet Ali Aydınlar University, 34638 Istanbul, Türkiye; ozgeumur00@gmail.com (Ö.U.); agopcitak@hotmail.com (A.Ç.); 5 Department of Pediatrics, Division of Pediatric Intensive Care, Istanbul Medeniyet University Prof. Dr. Süleyman Yalçın City Hospital, 34722 Istanbul, Türkiye; drmuhteremduyu@gmail.com (M.D.); drayseasik@gmail.com (A.A.); 6 Department of Pediatric Intensive Care, Bağcılar Training and Research Hospital, University of Health Sciences, 34303 Istanbul, Türkiye; abdulrahman.ozel@saglik.gov.tr

**Keywords:** children, influenza A virus, influenza B virus, pediatric intensive care unit, respiratory syncytial virus, sepsis, vaccination coverage, viral co-infection

## Abstract

**Highlights:**

**What are the main findings?**

• Two distinct severe influenza phenotypes emerged among children in the post-COVID-19 era: a respiratory-dominant form linked to Influenza A + RSV co-infection and a neuroinflammatory form associated with Influenza B.
• Influenza B infection independently predicted sepsis and neurological complications, while RSV co-infection drove early respiratory failure in infants.

**What are the implications of the main findings?**

• Post-pandemic “immunity gap” and extremely low influenza vaccination rates (2–4%) appear to amplify disease severity and PICU admissions in children.
• Strengthening pediatric influenza and RSV immunization policies is urgently required to reduce morbidity and mortality in future respiratory virus seasons.

**Abstract:**

**Background**: Severe pediatric influenza remains a major clinical burden, yet its phenotype in the post-COVID-19 period has not been fully characterized. The pandemic’s infection-control measures created an “immunity gap” among children, altering viral epidemiology and severity. This multicenter study from Türkiye defines the clinical spectrum and outcomes of influenza cases requiring intensive care, providing one of the first regional datasets after the pandemic. **Methods**: We retrospectively analyzed 85 children with influenza admitted to five tertiary PICUs in İstanbul between 2024 and 2025. Demographics, clinical features and outcomes were compared across groups. Predictors of sepsis, pediatric ARDS, and mechanical ventilation were identified through multivariate logistic regression. **Results**: Influenza A + RSV co-infection occurred in 14% and affected significantly younger infants, presenting with more severe respiratory distress, hypoxemia, and bronchiolitis. Influenza B was associated with distinct neurotropic features—encephalopathy and seizures in 48%—and a higher risk of sepsis (OR 3.27, 95% CI 1.02–10.53). Hypoxemia, elevated vasoactive–inotropic score, and high PaCO_2_ independently predicted mechanical ventilation and poor outcomes. Only 2–4% of children had received influenza vaccination. **Conclusions**: This multicenter analysis reveals a post-pandemic surge of severe pediatric influenza characterized by dual respiratory and neurological phenotypes. RSV co-infection drives early respiratory failure in infants, whereas Influenza B carries a disproportionate risk of neuroinflammation and sepsis. The study provides evidence from Türkiye that the post-COVID “immunity gap” and critically low vaccination coverage contribute to increased PICU admissions. Strengthening pediatric influenza immunization and RSV prevention policies is urgently warranted to mitigate these outcomes.

## 1. Introduction

Influenza remains one of the leading causes of acute respiratory illness and hospitalization among children worldwide [1]. Although most pediatric infections are self-limiting, severe cases may progress to respiratory failure, neurological complications, or even death [2]. Influenza A and B viruses are the principal pathogens responsible for annual seasonal epidemics, with Influenza A subtypes H1N1 and H3N2 historically linked to major global outbreaks [3]. Despite the widespread availability of vaccines and antiviral therapy, influenza continues to impose a substantial burden on pediatric health—particularly among infants and young children with comorbidities or limited prior exposure. Although most pediatric influenza cases are mild, a subset can progress to severe respiratory failure, neurological complications, or even death [4]. The COVID-19 pandemic profoundly altered the circulation of respiratory viruses through widespread infection control measures, resulting in a period of markedly reduced viral exposure—often described as an “immunity gap”—among young children. Consequently, recent influenza seasons have demonstrated a shift toward more severe clinical presentations, particularly in children lacking prior natural immunity [4].

Coinfection with respiratory syncytial virus (RSV) and infection with specific influenza types, especially Influenza B, have been associated with more complicated disease courses, including bronchiolitis, encephalopathy, and sepsis [5]. Early recognition of these high-risk patterns is essential for optimizing intensive care management and preventing adverse outcomes. However, national data from Türkiye regarding severe influenza cases requiring pediatric intensive care in the post-COVID-19 era remain limited [5,6].

This multicenter study investigates the clinical features, complications, and outcomes of children admitted to pediatric intensive care units (PICU) with laboratory-confirmed influenza during the 2024–2025 season in Türkiye. The study also aims to identify risk factors associated with severe disease and mortality, emphasizing the urgent public health need to improve vaccination coverage and strengthen surveillance systems [6].

## 2. Materials and Methods

### 2.1. Study Population

This multicenter retrospective study included children aged 1 month to 18 years who were admitted to five PICUs in İstanbul, Türkiye. All cases were identified retrospectively through hospital microbiology databases and institutional electronic medical records, based on real-time reverse transcriptase–polymerase chain reaction (RT-PCR)-confirmed influenza infections between 1 September 2024 and 30 April 2025. All eligible consecutive admissions were included without exclusions.

### 2.2. Data Collection

For each patient, demographic information, clinical presentation, comorbidities, laboratory and microbiologic findings, radiologic results, vaccination status, treatments, response to therapy, and outcomes were recorded. Data regarding noninvasive respiratory support and invasive mechanical ventilation (MV) were also collected.

Respiratory samples were collected at admission using nasopharyngeal swabs in all cases. In ventilated patients, tracheal aspirates or BAL were obtained where clinically indicated. Only the first sample was used for diagnostic inclusion. All diagnoses were based on multiplex RT-PCR; no antigen-based testing was used.

Influenza vaccination status was obtained from patient medical records and national immunization tracking systems. All vaccinations were from the current 2024–2025 season. In comparison, national influenza vaccine uptake among Turkish children for this period was estimated at approximately 6–8%.

The severity of pediatric acute respiratory distress syndrome (PARDS) was categorized as mild, moderate, or severe according to the Second Pediatric Acute Lung Injury Consensus Conference criteria. Sepsis was defined using the Phoenix Sepsis Score, while influenza-associated encephalopathy (IAE)—encompassing seizures, encephalitis, encephalopathy, Reye syndrome, and other neurologic complications—was defined according to the Centers for Disease Control and Prevention (CDC) criteria.

Respiratory specimens were analyzed using the Bio-Speedy RT-PCR kit (Bioeksen, Istanbul, Türkiye) on the CFX96 Touch real-time PCR platform (Bio-Rad Laboratories, Mannheim, Germany). This assay simultaneously detects multiple respiratory pathogens, including adenovirus, human coronaviruses (OC43, NL63, HKU1, 229E), human metapneumovirus, human rhinovirus/enterovirus, influenza A and B viruses, *Mycoplasma pneumoniae*, parainfluenza viruses 1–4, respiratory syncytial virus (RSV) A/B, severe acute respiratory syndrome coronavirus 2 (SARS-CoV-2), human parechovirus, *Haemophilus influenzae*, and *Streptococcus pneumoniae*.

### 2.3. Definitions

Sepsis was defined according to the Phoenix Sepsis Score, requiring evidence of systemic inflammatory response and suspected/proven infection. Influenza-associated encephalopathy (IAE) was defined per CDC criteria, including acute onset of altered mental status, seizures, or encephalitis without alternative explanation.

Cause of death was determined by treating PICU teams based on the clinical course, available microbiologic data, and consensus documentation in discharge or mortality summaries (e.g., primary viral pneumonia, secondary bacterial sepsis).

### 2.4. Statistical Analysis

All analyses were performed using IBM SPSS Statistics for Mac, Version 30.0 (IBM Corp., Armonk, NY, USA). The Shapiro–Wilk test was applied to assess normality. Continuous variables were expressed as median and interquartile range (IQR) and compared using the Kruskal–Wallis test. Categorical variables were expressed as frequencies and percentages and compared using Pearson’s chi-square or Fisher’s exact test, as appropriate. Univariate logistic regression analyses were conducted to identify factors potentially associated with adverse outcomes, including mortality, prolonged PICU stay, PARDS, neurological complications, and need for mechanical ventilation. Variables with *p* < 0.10 in univariate analyses were entered into multivariate logistic regression models using a backward (likelihood ratio) approach. Odds ratios (ORs) with 95% confidence intervals (CIs) were reported. Model fit was evaluated using the Hosmer–Lemeshow goodness-of-fit test. A two-tailed *p* value < 0.05 was considered statistically significant. Comparisons among infection groups (Influenza A, Influenza B, and Influenza A + RSV) were performed separately for demographic, clinical, and laboratory characteristics, interventions, and outcomes. Significant findings are presented in the respective tables.

### 2.5. Ethical Approval

The study protocol was approved by the Ethics Committee of Acıbadem Mehmet Ali Aydınlar University School of Medicine (Approval Date: 2 October 2025, No.: 2025-15/225). The research was conducted in accordance with the ethical principles outlined in the Declaration of Helsinki. The requirement for informed consent was waived due to the retrospective nature of the study.

### 2.6. Patient Characteristics

A total of 85 pediatric patients with laboratory-confirmed influenza infection were included in the study. Among them, 50 (58.8%) had Influenza A, 23 (27.1%) had Influenza B, and 12 (14.1%) had Influenza A plus RSV co-infection. Overall, 47 patients (55%) were male and 38 (45%) were female.

## 3. Result

### 3.1. Comparison Between Influenza A, Influenza B, and Influenza A + RSV Groups

Children with Influenza A + RSV co-infection were significantly younger than those with Influenza A or Influenza B (median age, 2 [1.4–6] months vs. 31 [7–60] months and 38 [20–87] months, respectively; *p* < 0.001). Body weight was correspondingly lowest in the co-infected group (*p* < 0.001).

Respiratory findings were more prominent among co-infected patients. Rates of tachypnea, tachycardia, hypoxemia, and respiratory distress were significantly higher (100%) in the Influenza A + RSV group compared with those infected with Influenza A or B alone. Wheezing and crepitations were also more frequent in this cohort. Notably, poor feeding was observed in all children with co-infection and differed significantly across groups (*p* = 0.010).

Conversely, neurological manifestations such as encephalopathy (*p* = 0.012) and seizures (*p* = 0.014) occurred predominantly among children with Influenza B. Other clinical symptoms, including fever, cough, vomiting, and diarrhea, did not differ significantly among the three groups. Detailed demographic and clinical characteristics are summarized in Table 1.

### 3.2. Comorbidities, PICU Interventions, and Complications

Comorbid conditions and treatment interventions are summarized in Table 2. Bronchiolitis was significantly more common in the Influenza A + RSV group (91.7%) compared with the Influenza A (32%) and Influenza B (13%) groups (*p* < 0.001). In contrast, sepsis was observed more frequently in children with Influenza B than in the other groups (*p* = 0.054).

Regarding PICU management, oxygen therapy, intravenous corticosteroids, and bronchodilator use (salbutamol and ipratropium bromide) were significantly more frequent among patients with Influenza A + RSV co-infection (*p* = 0.038, *p* = 0.048, *p* = 0.006, and *p* < 0.001, respectively). These findings indicate that RSV co-infection was associated with a greater need for respiratory support and anti-inflammatory treatment.

Among complications, respiratory failure occurred in 83.3% of children with Influenza A + RSV co-infection, significantly higher than in those with Influenza A (38%) or Influenza B (39%) (*p* = 0.015). Neurological complications were seen predominantly in the Influenza B group (47.8%), whereas none were observed among co-infected patients (*p* = 0.001). Other complications—including ARDS, acute kidney injury (AKI), and heart failure—did not differ significantly across groups.

### 3.3. Laboratory and Blood Gas Analysis

Laboratory parameters differed significantly among the three groups (Table 3). Leukocyte and hemoglobin values were similar across groups, whereas platelet counts varied significantly, with the highest values observed in children with Influenza A + RSV co-infection (*p* < 0.001 for lowest and *p* = 0.047 for highest counts).

Inflammatory markers also showed group-specific differences. Median C-reactive protein (CRP) and procalcitonin (PCT) levels were significantly higher in patients with Influenza B compared with those with Influenza A or Influenza A + RSV co-infection (*p* = 0.048 and *p* = 0.023, respectively), suggesting a more pronounced systemic inflammatory response. No significant intergroup differences were observed for lactate, creatinine, liver enzymes (AST, ALT), or LDH values.

At admission (T0), median pH values differed significantly among groups (*p* = 0.048), while PaO_2_, PaCO_2_, HCO_3_^−^, and lactate levels were comparable. After 24 h (T24), all blood gas parameters—including pH, PaO_2_, PaCO_2_, HCO_3_^−^, and lactate—remained similar across the three groups.

### 3.4. Comparison Between Patients with Influenza A and Influenza B (Excluding RSV Co-Infection)

After excluding patients with RSV co-infection, subgroup analysis included 50 children with Influenza A and 23 with Influenza B infection. No significant differences were found in demographic characteristics or in the frequency of clinical symptoms such as fever, tachypnea, and other presenting signs. However, tachycardia during hospitalization was observed more frequently in the Influenza A group (80.0%) than in the Influenza B group (52.2%; *p* = 0.017). The median durations of hospitalization and PICU stay were similar between groups (*p* = 0.532 and *p* = 0.350, respectively).

Among laboratory parameters, peak leukocyte counts were significantly higher in patients with Influenza A compared with those with Influenza B (15,100 ± 8107 vs. 11,125 ± 5109; *p* = 0.014), while other hematologic and biochemical measures did not differ significantly.

In terms of complications, sepsis occurred more often in children with Influenza B (52.2%) than in those with Influenza A (28.0%; *p* = 0.042). Severe acute respiratory distress syndrome (ARDS) was also more frequent in the Influenza B group (39.1% vs. 12.0%; *p* = 0.011). Neurological complications were markedly higher among children with Influenza B (47.8% vs. 16.0%; *p* = 0.006). Mortality was higher in the Influenza B group (13.0%) compared with the Influenza A group (6.0%), although this difference did not reach statistical significance (*p* = 0.278).

### 3.5. Mortality-Associated Clinical and Treatment Factors

Overall mortality was 7% (6 of 85 patients), including three deaths among children with Influenza A and three with Influenza B; no deaths occurred in those with RSV co-infection.

At presentation, mortality was significantly higher among children with apnea (20.0% vs. 4.3%; *p* = 0.031), cyanosis (21.1% vs. 2.9%; *p* = 0.007), and poor feeding (*p* = 0.047). During hospitalization, the development of apnea (18.8% vs. 4.2%; *p* = 0.043), cyanosis (33.3% vs. 2.7%; *p* < 0.001), or encephalopathy (16.0% vs. 3.3%; *p* = 0.038) was also associated with increased mortality. All patients who developed sepsis had a significantly higher risk of death (21.4% vs. 0%; *p* < 0.001).

Mortality was particularly elevated among children with comorbid conditions such as neuromotor disorders (*p* = 0.007), congenital heart disease (*p* = 0.037), chronic lung disease (*p* = 0.003), hematologic disorders (*p* = 0.016), and genetic syndromes (*p* = 0.010).

Indicators of critical illness strongly correlated with mortality. A vasoactive–inotropic score (VIS) ≥ 200 was a direct predictor of death (*p* < 0.001). The need for continuous renal replacement therapy (CRRT) (50.0% vs. 4.7%; *p* < 0.001) and the presence of pediatric ARDS (33.3% vs. 3.8%; *p* = 0.001) were also independently associated with death. All patients with septic shock (21.4% vs. 0%; *p* < 0.001) and nearly half of those with acute kidney injury (42.9% vs. 3.7%; *p* < 0.001) did not survive. Heart failure occurred in five patients, four of whom died (*p* < 0.001), while one patient with air-leak syndrome also succumbed.

Among therapeutic interventions, both invasive mechanical ventilation (26.3% vs. 1.5%; *p* < 0.001) and inotropic or vasopressor support (60.0% vs. 0%; *p* < 0.001) were significantly associated with mortality.

### 3.6. Predictors of Sepsis, Pediatric ARDS, and Mechanical Ventilation

Multivariate logistic regression analyses were conducted to identify independent predictors of sepsis, PARDS, and the need for mechanical ventilation (Table 4).

Lower serum albumin levels and higher C-reactive protein (CRP) concentrations were independently associated with sepsis (OR = 0.24, 95% CI 0.09–0.62, *p* = 0.003; OR = 1.013, 95% CI 1.001–1.024, *p* = 0.027, respectively). Children with Influenza B infection were also more likely to develop sepsis compared with those with Influenza A (OR = 3.27, 95% CI 1.02–10.53, *p* = 0.047).

Baseline hypoxemia (SpO_2_ < 92%) and older age independently predicted the development of PARDS (OR = 17.1, 95% CI 1.39–211.3, *p* = 0.027; OR = 1.020 per month, 95% CI 1.006–1.033, *p* = 0.004).

The need for mechanical ventilation was significantly associated with higher vasoactive–inotropic scores (VIS) (OR = 1.021 per point, 95% CI 1.004–1.038, *p* = 0.013) and elevated PaCO_2_ at admission (OR = 1.075 per mmHg, 95% CI 1.003–1.152, *p* = 0.042).

All regression models demonstrated good calibration according to the Hosmer–Lemeshow test (*p* > 0.05) and acceptable discriminative performance (AUC > 0.75).

Model AUCs were 0.79 for sepsis, 0.82 for PARDS, and 0.85 for mechanical ventilation. Sample sizes were *n* = 85 for all models, with 26 sepsis cases, 9 PARDS cases, and 19 ventilated cases. While PARDS model events were relatively limited, the Hosmer–Lemeshow test indicated good calibration.

## 4. Discussion

This multicenter study provides one of the most comprehensive descriptions to date of severe pediatric influenza requiring intensive care in Türkiye during the 2024–2025 season. The findings delineate distinct clinical patterns among Influenza A, Influenza B, and Influenza A + RSV co-infection, emphasizing the ongoing effects of the post-COVID-19 “immunity gap,” persistently low influenza vaccination rates, and unique morbidity and mortality profiles in critically ill children [7,8,9].

### 4.1. Severe Respiratory Manifestations Associated with RSV Co-Infection

Children with Influenza A + RSV co-infection were significantly younger and demonstrated the highest rates of respiratory distress, tachypnea, hypoxemia, and bronchiolitis. These patients frequently required escalated respiratory support, including supplemental oxygen, bronchodilators, and corticosteroids. The synergistic interaction between RSV and influenza likely accounts for the observed severity, as RSV promotes epithelial injury, airway edema, and mucus plugging, thereby amplifying lower respiratory tract involvement. Consistent with previous studies, infants coinfected with RSV and influenza exhibit higher rates of PICU admission, respiratory failure, and need for mechanical ventilation compared with single-virus infections [10,11,12,13]. Our results reinforce the importance of early recognition and aggressive respiratory management in coinfected infants to prevent rapid decompensation.

The increased use of bronchodilators and steroids in RSV co-infected patients may partially reflect clinician response to wheezing rather than illness severity per se, in line with existing literature on RSV bronchiolitis treatment.

### 4.2. Influenza B and Its Neurotropic Signature: A Distinct Risk Profile

A striking observation in this cohort was the disproportionately high rate of neurological complications among children with Influenza B infection—nearly half developed encephalopathy or seizures—despite comparable demographics and initial presentations to those with Influenza A. Although the difference in mortality did not reach statistical significance, the higher death rate in the Influenza B group suggests a potentially greater neurovirulent capacity of this subtype. These results support previous evidence indicating that Influenza B may exhibit enhanced neurotropism and should be recognized as an important cause of acute neurologic injury in pediatric populations [8,14,15].

Furthermore, children with Influenza B demonstrated higher levels of CRP and procalcitonin and were more likely to develop sepsis, indicating a stronger systemic inflammatory response compared with Influenza A. This finding aligns with prior reports showing that Influenza B elicits greater cytokine activation and is more frequently associated with bacterial co-infection and sepsis, which may contribute to its elevated inflammatory biomarker profile [8,16].

### 4.3. Determinants of Poor Clinical Outcomes and Mortality

Although overall mortality in this cohort was relatively low (7%), several clinical and laboratory parameters emerged as strong predictors of death or a prolonged intensive care course. Children who developed apnea, cyanosis, sepsis, neurological impairment, or multi-organ dysfunction were more likely to require vasoactive support, continuous renal replacement therapy (CRRT), or mechanical ventilation. Elevated PaCO_2_, baseline hypoxemia, and high vasoactive–inotropic scores (VIS) were independently associated with mechanical ventilation, reflecting combined respiratory and circulatory compromise. These observations are consistent with prior pediatric critical-care reports demonstrating that derangements in gas exchange and perfusion parameters reliably predict adverse outcomes [17].

Neurological involvement also appeared as an important marker of disease severity beyond the respiratory system. In a retrospective series of 348 children with influenza, neurological manifestations such as seizures or encephalopathy were significantly more frequent among fatal cases (44.4%; *p* = 0.02) [17]. Similarly, other studies have identified acute respiratory distress syndrome (ARDS), elevated D-dimer, and acute necrotizing encephalopathy (ANE) as independent predictors of mortality in pediatric influenza [16].

The prognostic role of the VIS in critically ill children is increasingly recognized. In a large cohort of pediatric septic shock, the median cumulative VIS at 96 h was 15 (IQR: 8–26), indicating moderate cardiovascular support requirements and was strongly correlated with mortality and length of PICU stay [18]. Another investigation demonstrated that VIS values exceeding 70 were predictive of treatment failure or death in pediatric patients requiring intensive support [19]. Our findings are concordant with these data, confirming VIS as a simple yet powerful marker of circulatory failure and overall illness severity.

Hypoxemia and hypercapnia (elevated PaCO_2_) at admission indicate advanced respiratory compromise. Influenza-associated PARDS carries mortality rates up to 30–35% when severe hypoxemia is present [20]. The need for CRRT and the occurrence of acute kidney injury (AKI) in our cohort further illustrate the multisystemic nature of severe influenza. This aligns with prior literature showing that viral infections complicated by shock or AKI portend worse outcomes and necessitate early organ-support interventions [21,22].

Neurological dysfunction—including encephalopathy and seizures—was one of the most critical prognostic indicators. A multicenter U.S. surveillance study identified influenza-associated encephalopathy (IAE) in approximately 9% of pediatric influenza deaths, rising to 13% in preliminary 2024–2025 data [3]. Nearly all affected children required mechanical ventilation (93%) and had markedly high fatality rates. These findings emphasize that neurological involvement signals an unfavorable trajectory, underscoring the need for early neuroimaging, close neurologic monitoring, and prompt antiviral therapy when neurological signs appear.

### 4.4. Pediatric ARDS Risk and Phenotypic Differences

The incidence of PARDS among children with severe influenza varies considerably according to viral subtype and age group. In a multicenter study, influenza-associated PARDS was reported in predominantly young children (mean age ≈ 64 months), with nearly 60% of cases occurring in those under 5 years of age and an associated mortality of 33% [20]. Another review of viral triggers of ARDS found that influenza and respiratory syncytial virus (RSV) infections tend to provoke more severe lung injury in older children compared with the classic RSV-predominant phenotype seen in infancy, possibly reflecting age-related differences in lung compliance, immune maturation, and co-infection dynamics [23].

Consistent with these observations, older age in our cohort was independently associated with a higher risk of PARDS, whereas younger infants—most frequently those with RSV co-infection—were more likely to exhibit bronchiolitis or non-parenchymal respiratory distress. These findings highlight that the clinical phenotype of viral lung injury differs across developmental stages. Early implementation of lung-protective ventilation strategies, particularly in older children presenting with hypoxemia or hypercapnia, may help mitigate the progression to PARDS and improve outcomes.

### 4.5. A Post-COVID-19 Era Surge Driven by the “Immunity Gap”

Following the introduction of widespread nonpharmaceutical interventions (NPIs) during the COVID-19 pandemic—including school closures, masking, and physical distancing—the circulation of seasonal respiratory viruses such as influenza A/B and RSV decreased dramatically. As a consequence, serologic and epidemiologic data from multiple countries now document a measurable decline in population-level immunity. For example, a 2024 study from Norway demonstrated significantly lower antibody titers against influenza A(H1N1)pdm09 and A(H3N2) in sera collected during 2021–2022 compared with 2019, confirming a tangible “immunity gap” in young children [24]. Similarly, surveillance data from Korea showed that the proportion of older children (7–18 years) hospitalized for influenza nearly doubled in post-pandemic seasons compared with pre-pandemic years (14% vs. 7%), reflecting a shift in age distribution toward previously unexposed cohorts [25].

Our findings parallel these global observations. The youngest children in our cohort—those born or raised during the pandemic—experienced more severe illness when co-infected with influenza and RSV, consistent with the hypothesis of accumulated susceptibility or “immunity debt” [26]. This concept suggests that reduced exposure to circulating respiratory viruses during the pandemic led to a transient increase in immunologic naïveté, amplifying the severity of subsequent epidemics once normal circulation resumed. It is plausible that a similar immunologic shift occurred in Türkiye, contributing to the observed rise in both the frequency and severity of pediatric influenza cases [27].

Together, these results reinforce the urgent need to strengthen pediatric influenza vaccination programs, re-establish routine viral surveillance, and promote early-season immunization to close the post-pandemic immunity gap and protect vulnerable populations.

While older children in our cohort may reflect the effects of reduced viral exposure during the pandemic (‘immunity gap’), infants born after 2022 were not directly impacted by such measures. Therefore, their increased severity likely reflects baseline vulnerability and RSV virulence rather than pandemic-associated immunity debt.

### 4.6. Dangerously Low Vaccination Coverage: A National Public Health Priority

Alarmingly, only 2–4% of children in our cohort had received the influenza vaccine, reflecting critically low vaccination coverage that likely contributed to the frequency and severity of cases requiring intensive care. In comparison, national pediatric influenza vaccination coverage in Türkiye during the 2024–2025 season was estimated at 6–8%, indicating that vaccine uptake in our cohort was even lower than national averages. Prior studies have shown that seasonal influenza vaccination can reduce the risk of PICU admission among children by up to 74% in some seasons, and vaccine effectiveness for severe outcomes—including ICU admission—remains above 50% even during years of suboptimal strain match [28].

While maternal vaccination against influenza is recommended in Türkiye, uptake remains low and maternal immunization data were not captured in this dataset.

These findings highlight an urgent need to strengthen national influenza immunization strategies. Expanding vaccine coverage, prioritizing high-risk pediatric groups such as infants and children with neurological or congenital heart disease, and enhancing pediatrician-led advocacy and parental education should be national priorities. Integrating annual influenza vaccination reminders into routine pediatric immunization schedules would help increase uptake. Furthermore, given the significant burden of RSV co-infection identified in this study, the development and widespread adoption of RSV immunization strategies could further mitigate severe respiratory outcomes in young children.

While our findings highlight associations between influenza subtypes and clinical outcomes, causality cannot be definitively established. Potential confounding factors such as underlying comorbidities may have influenced observed outcomes. Additionally, as our cohort only included children admitted to tertiary PICUs, selection bias toward more severe cases may exist. Long-term neurologic outcomes were not available and represent a limitation of our retrospective design.

### 4.7. Strengths and Limitations

This study has several strengths. It represents one of the first multicenter investigations from Türkiye to characterize severe pediatric influenza requiring intensive care in the post-COVID-19 era, providing nationally relevant epidemiologic and clinical insights across multiple tertiary PICUs. The inclusion of laboratory-confirmed cases, detailed subgroup analyses, and multivariate modeling adds robustness to the findings.

However, certain limitations must be acknowledged. The retrospective design introduces potential information bias, and long-term neurological outcomes were not assessed. Additionally, the relatively small number of deaths limits the precision of mortality-related estimates. Despite these constraints, the multicenter design and diverse patient population enhance the generalizability of the results and contribute valuable data to the evolving global understanding of severe pediatric influenza.

## 5. Conclusions

In conclusion, severe pediatric influenza continues to impose a significant clinical and public health burden in the post-COVID-19 era. RSV co-infection was associated with more severe respiratory failure among younger children, whereas Influenza B was more frequently linked to neurological complications and sepsis. Hypoxemia, elevated vasoactive–inotropic scores, and multi-organ dysfunction emerged as key warning indicators of poor outcomes. The strikingly low influenza vaccination coverage observed underscores the urgent need to strengthen national immunization efforts and implement RSV prevention strategies. Together, these measures are essential to reduce the severity and frequency of future pediatric influenza outbreaks and to protect the most vulnerable children.

## Figures and Tables

**Table 1:** Comparison of demographic and clinical characteristics among children with Influenza A, Influenza B, and Influenza A + RSV co-infection.

Variable	Influenza A (*n* = 50)	Influenza B (*n* = 23)	Influenza A + RSV (*n* = 12)	*p* Value
Age, months, median (IQR)	31 (7–60)	38 (20–87)	2 (1.4–6)	**<0.001 ^1^**
Sex, male, *n* (%)	27 (54%)	14 (60.9%)	6 (50%)	0.795 ^2^
Weight, kg, median (IQR)	12.6 (8–21)	15 (10–25)	5.1 (4.4–6.5)	**<0.001 ^1^**
Age of mother, years, median (IQR)	32 (29–36)	35 (28–40)	32 (27–36)	0.530 ^1^
Duration of breastfeeding, months, median (IQR)	6 (2–12)	7.5 (2–13)	2 (1.5–2)	0.236 ^1^
Smoker mother, *n* (%)	19 (38%)	7 (30%)	1 (8%)	0.138 ^2^
Hospitalization duration, days, median (IQR)	5 (3–8)	5.5 (4–8)	4.5 (3–5)	0.062 ^1^
Vaccinated against influenza, *n* (%)	1 (2%)	1 (4.3%)	0 (0%)	0.700
Clinical Symptoms, *n* (%)				
Fever, *n* (%)	38 (76%)	19 (82.6%)	8 (66.7%)	0.569 ^2^
Tachycardia, *n*(%)	37 (74%)	13 (56.5%)	12 (100%)	**0.022 ^2^**
Poor feeding, *n* (%)	36 (72%)	14 (60.9%)	12 (100%)	**0.010 ^2^**
Cough, *n* (%)	33 (66%)	15 (65.2%)	11 (91.7%)	0.196 ^2^
Respiratory distress, *n* (%)	33 (66%)	12 (52.2%)	12 (100%)	**0.016 ^2^**
Nasal congestion, *n* (%)	31 (62%)	13 (56.5%)	10 (83.3%)	0.277 ^2^
Tachypnea, *n* (%)	32 (64%)	12 (52.2%)	12 (100%)	**0.004 ^2^**
Hypoxemia (SpO_2_ < 92%), *n* (%)	30 (60%)	12 (52.2%)	12 (100%)	**0.015 ^2^**
Crepitations, *n* (%)	21 (42%)	10 (43.5%)	10 (83.3%)	**0.032 ^2^**
Vomiting, *n* (%)	18 (36%)	10 (43.5%)	3 (25%)	0.556 ^2^
Wheezing, *n* (%)	18 (36%)	5 (21.7%)	10 (83.3%)	**0.002 ^2^**
Encephalopathy, *n* (%)	16 (32%)	8 (34.8%)	0 (0%)	**0.012 ^2^**
Seizures, *n* (%)	15 (30%)	11 (47.8%)	0 (0%)	**0.014 ^2^**
Diarrhea, *n* (%)	12 (24%)	4 (17.4%)	1 (8.3%)	0.445 ^2^
Cyanosis, *n* (%)	11 (22%)	4 (17.4%)	4 (33.3%)	0.559 ^2^
Apnea, *n* (%)	7 (14%)	4 (17.4%)	4 (33.3%)	0.288 ^2^
Focal lung findings (unilateral), *n* (%)	3 (6%)	1 (4.3%)	1 (8.3%)	0.892 ^2^

^1^ Kruskal–Wallis test, ^2^ Pearson chi-square test. Significant *p* values shown in bold.

**Table 2:** Comparison of comorbidities, PICU interventions, and complications among children with Influenza A, Influenza B, and Influenza A + RSV co-infection.

Variable	Influenza A (*n* = 50)	Influenza B (*n* = 23)	Influenza A + RSV (*n* = 12)	*p* Value
Comorbidities				
Bronchiolitis	16 (32%)	3 (13%)	11 (91.7%)	<0.001 *
Sepsis	14 (28%)	12 (52.2%)	2 (16.7%)	0.054
Pneumonia	12 (24%)	9 (39.1%)	3 (25%)	0.396
Neuromotor disease	12 (24%)	7 (30.4%)	0 (0%)	0.111
Genetic disorder	7 (14.3%)	4 (18.2%)	1 (8.3%)	0.736
Chronic kidney disease	6 (12%)	3 (13%)	1 (8.3%)	0.916
Reactive airway disease	5 (10%)	0 (0%)	0 (0%)	0.156
CNS infection	5 (10%)	4 (17.4%)	0 (0%)	0.278
Prematurity	5 (10%)	4 (17.4%)	3 (25%)	0.354
Congenital heart disease	5 (10%)	2 (8.7%)	1 (8.3%)	0.975
Asthma	4 (8%)	0 (0%)	0 (0%)	0.230
Metabolic disease	2 (4.1%)	0 (0%)	0 (0%)	0.491
Immunodeficiency	1 (2%)	1 (4.3%)	1 (8.3%)	0.548
Hematologic disorder	1 (2%)	1 (4.3%)	0 (0%)	0.700
PICU Treatments and Interventions				
Antibiotic use	48 (96%)	23 (100%)	12 (100%)	0.488
Oxygen via mask	29 (58%)	11 (47.8%)	11 (91.7%)	0.038 *
IV steroid	22 (44%)	11 (47.8%)	10 (83.3%)	0.048 *
Inhaled steroid	22 (44%)	9 (39.1%)	6 (50%)	0.823
Salbutamol	22 (44%)	9 (39.1%)	11 (91.7%)	0.006 *
Ipratropium bromide	21 (42%)	6 (26.1%)	11 (91.7%)	<0.001 *
HFNC	13 (26%)	5 (21.7%)	4 (33.3%)	0.758
NIV/BİPAP	10 (20%)	3 (13%)	3 (25%)	0.655
Mechanical Ventilation	11 (22.4%)	8 (34.8%)	0 (0%)	0.065
Prone positioning	8 (16%)	5 (21.7%)	1 (8.3%)	0.592
Inotrope use	7 (14%)	3 (13%)	0 (0%)	0.391
Nasal cannula	6 (12%)	6 (26.1%)	0 (0%)	0.087
Inhaled epinephrine	5 (10%)	4 (17.4%)	1 (8.3%)	0.610
Plasma exchange	2 (4.0%)	1 (4.3%)	0 (0%)	0.772
CRRT	2 (4%)	2 (8.7%)	0 (0%)	0.481
ECMO	1 (2%)	0 (0%)	0 (0%)	0.702
Complications				
Respiratory failure	19 (38%)	9 (39.1%)	10 (83.3%)	0.015 *
Septic shock	16 (32%)	11 (47.8%)	1 (8.3%)	0.060
Neurological complication	8 (16%)	11 (47.8%)	0 (0%)	0.001 *
ARDS	6 (12%)	3 (13%)	0 (0%)	0.433
AKI	4 (8%)	3 (13%)	0 (0%)	0.410
Heart failure	2 (4.1%)	3 (13.6%)	0 (0%)	0.188

Pearson chi-square test was used. Significant *p* values shown in bold and with asterix *. CNS, central nervous system; HFNC, high-flow nasal cannula; NIV, non-invasive; CRRT, continuous renal replacement treatment; ECMO, extracorporeal membrane oxygenation; ARDS, acute respiratory distress syndrome; AKI, acute kidney injury.

**Table 3:** Comparison of laboratory and arterial blood gas parameters among children with Influenza A, Influenza B, and Influenza A + RSV co-infection.

Variable, Median (IQR)	Influenza A (*n* = 50)	Influenza B (*n* = 23)	Influenza A + RSV (*n* = 12)	*p* Value
Leukocyte (/mm^3^) (lowest)	5.56 (3.69–7.74)	5.38 (3.95–6.82)	7.2 (5.135–9.045)	0.207
Leukocyte (/mm^3^) (highest)	13.46 (9.3–19.88)	10.02 (8.28–13.77)	12.155 (10.76–13.575)	0.110
Hemoglobin (g/dL) (lowest)	9.7 (8.7–11)	9.1 (7.2–10.1)	8.6 (7.5–10.6)	0.224
Platelet (/mm^3^) (lowest)	172,500 (127,000–242,000)	172,000 (123,000–226,000)	317,000 (271,000–399,500)	<0.001 *
Platelet (/mm^3^) (highest)	403,500 (343,000–481,000)	343,000 (236,000–451,000)	497,000 (385,000–688,500)	0.047 *
AST (U/L) (highest)	53.5 (36–86)	48 (36–107)	42.0 (36.1–48)	0.258
ALT (U/L) (highest)	34 (21–65)	33 (21–79)	22 (19–41)	0.403
Creatinine (mg/dL) (highest)	0.44 (0.32–0.73)	0.44 (0.38–0.94)	0.34 (0.22–0.44)	0.115
LDH (U/L) (highest)	393 (317–601)	435 (329–609)	368 (312–414)	0.223
CRP (mg/L) (highest)	21 (2.64–66.83)	26.30 (7.94–72.3)	3.35 (0.45–30)	0.048 *
PCT (ng/mL) (highest)	1.51 (0.17–8.8)	1.55 (0.29–3.9)	0.15 (0.11–0.50)	0.023 *
Lactate (mmol/L) (highest)	3.2 (1.4–5.5)	2.3 (1.40–5.6)	4.10 (2.00–6.05)	0.575
PaCO_2_ (mmHg) (highest)	49.3 (44.0–53.9)	50.6 (43.7–55)	49.5 (45.1–54.8)	0.757
PaO_2_ (mmHg) (lowest)	46 (39–55)	43 (35–50)	38 (38–38)	0.348
Blood Gas Analysis at admission (T0)				
pH	7.32 (7.27–7.39)	7.37 (7.31–7.41)	7.32 (7.27–7.36)	0.048 *
PaO_2_ (mmHg)	78.30 (459.60–87.90)	74.35 (57.30–81.40)	73.30 (59–96.60)	0.324
PaCO_2_ (mmHg)	45.65 (40–50.2)	43.30 (40–50.6)	48.05 (43.60–51.15)	0.756
HCO_3_^−^ (mmol/L)	22.05 (20–23.7)	23.00 (22–26.2)	22.15 (20.40–23.85)	0.157
Lactate (mmol/L)	1.70 (1–2.90)	1.47 (1–2.1)	2 (1.55–3.45)	0.149
Blood Gas Analysis at 24th Hour (T24)				
pH	7.38 (7.36–7.42)	7.38 (7.36–7.42)	7.37 (7.31–7.42)	0.560
PaO_2_ (mmHg)	82 (77–93)	80 (70–91)	77.30 (59–91.3)	0.519
PaCO_2_ (mmHg)	38 (35.3–41.5)	41.20 (37.4–47.4)	38.70 (36–49.7)	0.173
HCO_3_^−^ (mmol/L)	23.1 (22–26.1)	23.70 (22.3–26)	22.70 (21.3–24)	0.363
Lactate (mmol/L)	1.5 (0.8–2.1)	1.20 (0.90–2.00)	1.90 (1.40–4.60)	0.107

Kruskal–Wallis test was used. Significant *p* values shown in bold and with an asterisk *. AST, Aspartate Aminotransferase; ALT, Alanine aminotransferase; LDH, lactate dehydrogenase; CRP, C-reactive protein; PCT, Procalcitonin; PaO_2_, Partial Arterial Oxygen Pressure; PaCO_2_, Partial Arterial Carbon Dioxide Pressure; HCO_3_^−^, Bicarbonate.

**Table 4:** Multivariate logistic regression analysis showing independent predictors of sepsis, pediatric ARDS, and the need for mechanical ventilation among children with influenza infection.

Outcome	Independent Predictor	OR	95% CI	*p* Value
Sepsis	Serum albumin (g/dL)	0.24	0.09–0.62	0.003 *
	C-reactive protein (mg/L)	1.013	1.001–1.024	0.027 *
	Influenza B (vs. A)	3.27	1.02–10.53	0.047 *
Pediatric ARDS	Hypoxemia (SpO_2_ < 92%)	17.1	1.39–211.3	0.027 *
	Age (per month)	1.020	1.006–1.033	0.004 *
Mechanical ventilation	VIS (per point)	1.021	1.004–1.038	0.013 *
	PaCO_2_ at admission (mmHg)	1.075	1.003–1.152	0.042 *

Hosmer–Lemeshow goodness-of-fit test *p* > 0.05 for all models, indicating adequate calibration. OR = odds ratio; CI = confidence interval. * indicates statistical significance.

## Data Availability

The data presented in this study are available on request from the corresponding author. The data are not publicly available due to privacy and ethical reasons.

## Abbreviations

AKI	Acute Kidney Injury
ALT	Alanine Aminotransferase
ANE	Acute Necrotizing Encephalopathy
ARDS	Acute Respiratory Distress Syndrome
AST	Aspartate Aminotransferase
CDC	Centers for Disease Control and Prevention
CI	Confidence Interval
COVID-19	Coronavirus Disease 2019
CRP	C-Reactive Protein
CRRT	Continuous Renal Replacement Therapy
ECMO	Extracorporeal Membrane Oxygenation
HCO_3_^−^	Bicarbonate
HFNC	High-Flow Nasal Cannula
IAE	Influenza-Associated Encephalopathy
IQR	Interquartile Range
LDH	Lactate Dehydrogenase
MV	Mechanical Ventilation
NPI	Nonpharmaceutical Intervention
NIV	Non-Invasive Ventilation
OR	Odds Ratio
PaCO_2_	Partial Arterial Carbon Dioxide Pressure
PaO_2_	Partial Arterial Oxygen Pressure
PARDS	Pediatric Acute Respiratory Distress Syndrome
PCR	Polymerase Chain Reaction
PICU	Pediatric Intensive Care Unit
PCT	Procalcitonin
RSV	Respiratory Syncytial Virus
RT-PCR	Real-Time Reverse Transcriptase Polymerase Chain Reaction
SARS-CoV-2	Severe Acute Respiratory Syndrome Coronavirus 2
SPSS	Statistical Package for the Social Sciences
VIS	Vasoactive–Inotropic Score
